# circSLC8A1 Acts as a Tumor Suppressor in Prostate Cancer via Sponging miR-21

**DOI:** 10.1155/2021/6614591

**Published:** 2021-04-01

**Authors:** Daoyuan Wang, Shuxian Yan, Lihui Wang, Yunlong Li, Baoping Qiao

**Affiliations:** ^1^Department of Urology, The First Affiliated Hospital of Zhengzhou University, Zhengzhou, China; ^2^Department of Urology, 989th Hospital of the Joint Logistic Support Force, Luoyang, China

## Abstract

**Background:**

There is more and more evidence showed that circRNAs played essentially role in the regulation of various biological processes. The role of circSLC8A1 in prostate cancer (PCa) is yet little known.

**Methods:**

The CircSLC8A1 expression in human prostate cancer was measured by qRT-PCR. The interplay between the specific circRNA, miRNA, and mRNA was investigated by RT-PCR and luciferase reporter assay. Through transient transfection of siRNA, the impacts of circSLC8A1 on PCa were discussed. Cell cycle evaluation, transwell assay, and CCK-8 assay were employed to determine its biological influences.

**Results:**

In this study, our data revealed that circSLC8A1 was downregulated in PCa tissues and cells. The reduction of circSLC8A1 resulted in the inhibition of cell proliferation and migration. In mechanism, circSLC8A1 exhibited a direct interaction with miR-21 and displayed as a miRNA sponge to inhibit PCa progression. The functional analysis revealed that the circSLC8A1/miR-21 axis may regulate the cell proliferation, angiogenesis, cell migration, epithelial to mesenchymal transition, MAPK signaling pathway, and chemokine signaling pathway.

**Conclusions:**

CircSLC8A1 functioned as an inhibitor of neoplasm via modulating the miR-21 and might serve as a prospective target for the treatment of PCa.

## 1. Background

The incidence rate of prostate cancer (PCa) ranked second amid all commonly occurred carcinomas, and the lethality of it was in fifth place among carcinoma-associated deaths in men worldwide [1]. As far as the treatment of PCa, radiotherapy is a commonly employed adjuvant treatment for surgery and chemotherapy, which could extend the dominant time for controlling carcinoma. Even though most of PCa stayed in the form of or inactivation for a long time, the metastasis progression perhaps contributed to poor prognostic status and more death. Herein, it is necessary to deeply elaborate the thorough molecular mechanisms related to PCa, which is conducive to uncovering new targets for diagnosis and treatment.

Circular RNAs (circRNAs) are a sort of ground substance that is not typically bound to the endogenously generated RNA [2, 3]. As a new class of endogenous noncoding RNA, circRNAs did not possess 5′ cap and 3′ poly covalently closed structure-like tail [4]. It is different from linear RNA, and circRNA is usually derived from reverse splicing event of an exon or intron. The reverse complementary sequence, containing the inverted repeat Alu pair and exon skipping, is essential for the formation of circRNA [5, 6]. Genome and transcriptome data generated from the next generation sequencing (NGS) projects and bioinformatic algorithms have screened and defined numerous circRNAs in eukaryotes [7–10], revealing that they are not just casual byproducts or “splicing noise.” High-throughput technology has been applied to in-depth characterize the identification and potential functions of circRNA [11, 12]. circRNA is a class of abundant and conserved RNA, existing widely in intricate tissues, cell types, or specific stages. Various circRNAs displayed importantly in carcinoma development [4, 13]. The differential expressed circular RNA in PCa was probably related to the resistance to enzalutamide [14]. Circfoxo3 facilitates PCa progression via sponging miR-29a-3p [15]. CircRNA-UCK2-caused increase expression of Tet1 would hinder PCa cell proliferation and invasion by sponging mirna-767-5p [16]. Increased expression of HOXB13 induced by the linkage of circular RNA ITCH and sponge-like miR-17-5p caused the suppression of PCa progression [17].

The circRNAs are emerging as a new class of noncoding RNAs implicated in multiple cancer types, and to explore the functional roles of circRNAs in PCa, we screened the circRNAs potentially involved in PCa by systematic literature review. In the present study, we found that the circSLC8A1 expression in the tissues and cells of PCa was dramatically reduced, and its expression displayed a positive correlation with the clinical DFS of PCa. Overall, our findings implied that CircSLC8A1 functioned as an inhibitor of neoplasm via modulating the miR-21, affording a prospective target for the treatment of PCa.

## 2. Methods

### 2.1. Public Gene Expression Dataset

The analysis of SLC8A1 expression levels in PCa was implemented in the GEPIA web server [18, 19], which curated all the cancer types of the Cancer Genome Atlas (TCGA) project.

### 2.2. Human Cell Lines and Tissues

Human PCa cell lines were obtained from SIBCB (Shanghai, China). PCa specimens and paired adjacent control specimens were collected from surgical patients in The First Affiliated Hospital of Zhengzhou University. All patients have autonomously signed written consent. Our experiments got approval of the ethics committee of our hospital. All tissues were stored in liquid nitrogen for long-term use.

### 2.3. Isolation and Quantation of RNA

Whole RNA was isolated by Trizol Reagent (TIANGEN, China) as manually described. RNA was reversely transcripted into the cDNA using Takara system (TIANGEN, China). AceQ qPCR SYBR Green Master Mix kit was employed to perform qRT- PCR on Roche 480. 2^-*ΔΔ*CT^ was carried to calculate the cycle threshold (CT) value of the normalized target gene expression [20].

### 2.4. Construction of siRNA

The sequences of siRNAs were acquired from GenePharma (Shanghai, China). Synthetic promiscuous siRNA was treated as scramble control. siRNAs were transfected into cells utilizing Lipofectamine 3000 (Invitrogen) referring to the instruction. Overall, RNA and protein were harvested at 48 hours posttransfection.

### 2.5. Transwell Assay

A density of 1 × 10^5^ cells in 500 *μ*L medium without FBS was plated in the upper chamber. Medium containing 10% FBS was added into the lower chamber as a chemical attractant. Cotton-tipped swabs were used to remove the cells staying in the upper chamber after culturing for the indicated time. The number of invaded or migrated cells at stochastic six fields was calculated.

### 2.6. Cell Proliferation Assay

CCK-8 kit (Dojindo, Japan) was utilized to determine the ability of PCa cell viability. Totally, 6,000 cells were plated in 96-well and then subjected to different treatments at the indicated time. The OD value of 450 nm was detected after incubation with 10 *μ*l CCK-8 solution for another 2 hours.

### 2.7. Luciferase Reporter Assay

Wild type cicrSLC8A1 sequence comprising the assumed binding site of miR-21 was inserted into the reporter vector. All final positive clones were verified by sequencing. Mutant cicrSLC8A1 sequence was also inserted into *t* reporter construct so as to examine the specific binding activity with miR-21. The luciferase activity was detected by luciferase assay kit (Promega, USA) at 24 hours posttransfection.

### 2.8. Statistical Analysis

All derived data were shown as the mean ± SD. Paired Student's *t*-test was introduced to assess the differences of the levels of circSLC8A1 and miR-21 in compared groups, and chi-square test was applied to determine the differences of more than two groups. The Pearson correlation coefficient analysis was also conducted [21]. A *p* value of <0.05 was thought to be statistically significant [22].

## 3. Results

### 3.1. CircSLC8A1 (hsa_circ_0000994) Is Obviously Reduced in PCa

**Figure 1:**
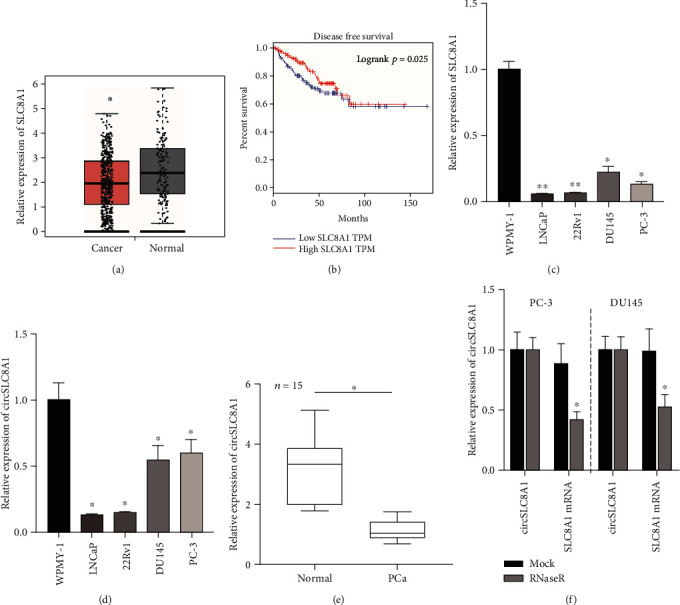
The circSLC8A1 expression is obviously reduced in PCa. (a) The TCGA dataset showed that SLC8A1 is significantly downregulated in PCa. (b) The circSLC8A1 expression exhibited an association with disease-free survival time in patients. (c) The SLC8A1 and (d) circSLC8A1 expression was found reduced in PCa cells. (e) The circSLC8A1 expression was found reduced in PCa tissues. (f) circSLC8A1 but not SLC8A1 was reduced by RNase *R*.

As shown in Figure 1(a), the linear RNA of circSLC8A1 was significantly downregulated in PCa compared to normal samples using the Cancer Genome Atlas (TCGA) dataset. Accordingly, the low expression of SLC8A1 was associated with shorter disease-free survival time in patients in the TCGA cohort (Figure 1(b)). Further investigation of SLC8A1 RNA products revealed that circSLC8A1 and SLC8A1 were reduced in PCa cells, including DU145, PC-3, 22Rv1, and LNCaP, in comparison with normal urothelial cells WPMY-1(Figures 1(c) and 1(d)). The analysis of circSLC8A1 in 15 paired PCa and normal prostate tissues revealed that circSLC8A1 was downregulated in PCa tissues (Figure 1(e)). Moreover, the incubation of RNase *R* led to a reduction in the level of SLC8A1 linear mRNA, not the levels of circSLC8A1 (Figure 1(f)), suggesting that circSLC8A1 was more stable than its linear RNA.

### 3.2. Knockdown of circSLC8A1 Enhanced the Proliferation and Migration

**Figure 2:**
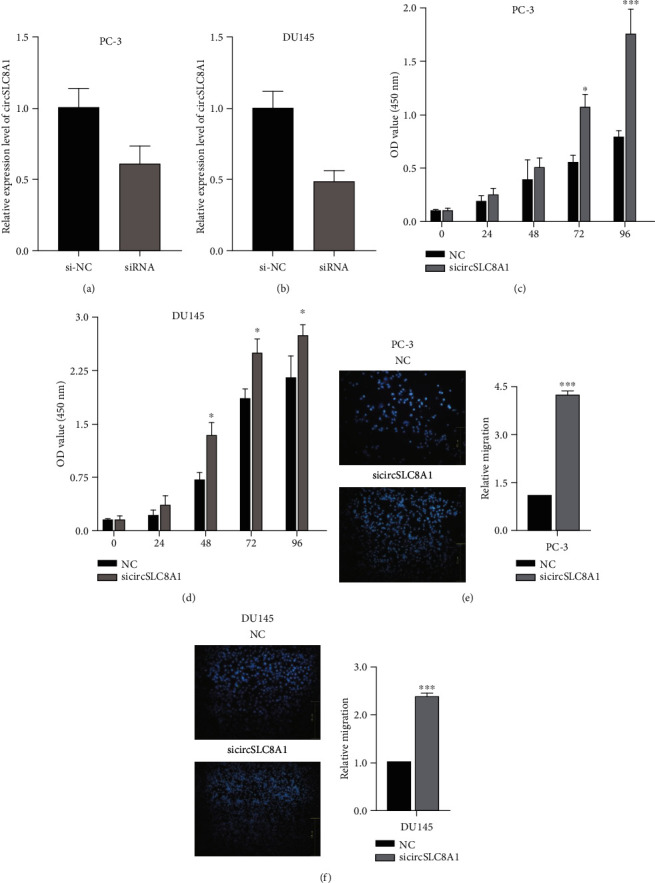
Knockdown of circSLC8A1 enhanced the migration and invasion. (a, b) circSLC8A1 was significantly decreased after transfected the sicircSLC8A1. (c, d) Knockdown of circSLC8A1 increases the proliferation capabilities. (e, f) Knockdown of circSLC8A1 increase the migration.

To further evaluate the functional impact of circSLC8A1 on PCa, we conducted functional assays to deeply investigate its functionalities. Firstly, we transfected the siRNA of circSLC8A1 into DU145 and PC-3 cells and observed that the circSLC8A1 expression was significantly reduced (Figures 2(a) and 2(b)). The CCK-8 assay showed that the proliferation capabilities of prostate cancer cells were dominantly enhanced by the knockdown of circSLC8A1 (Figures 2(c) and 2(d)). Transwell results showed that knockdown circSLC8A1 significantly increased migration of cells of DU145 and PC-3 (Figures 2(e) and 2(f)). These results indicated that circSLC8A1 exerted tumor-suppressing effect by inhibiting the proliferation and migration of PCa cells.

### 3.3. circSLC8A1 Functions as a Sponge for miR-21 in PCa Cells

**Figure 3:**
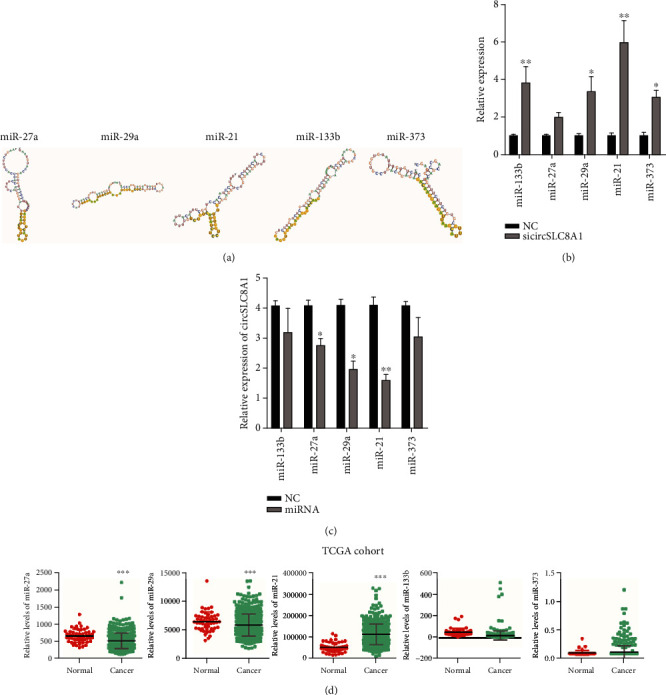
circSLC8A1 sponged miR-21 in PCa. (a)The interaction between miR-133b, miR-27a, miR-29a, miR-21 and miR-373, and circSLC8A1. (b) Knockdown of circSLC8A1 significantly induced the level of miR-133b, miR-29a, miR-21, and miR-373. (c) Overexpress miR-27a, miR-29a, and miR-21 reduced the expression of circSLC8A1. (d) The expression of miR-133b, miR-27a, miR-29a, miR-21, and miR-373 in PCa.

Previous reports have shown that circRNA played as a miRNA sponge to modulate the miRNA expression [23, 24]. To further explore whether circSLC8A1 could function as a miRNAs sponge in PCa cells, we employed two widely used prediction websites including circBANK and RegRNA 2.0. We selected 5 candidate miRNAs, including miR-133b, miR-27a, miR-29a, miR-21, and miR-373, which were predicted to bind to circSLC8A1 (Figure 3(a)). Subsequently, we knockdown circSLC8A1 and detected the effect of circSLC8A1 on these candidate miRNAs. It was confirmed that the knockdown of circSLC8A1 significantly increased the expression of miR-133b, miR-29a, miR-21, and miR-373 (Figure 3(b)). Moreover, we detected the circSLC8A1 levels after the overexpression of these miRNAs to confirm the circSLC8A1 sponge effect. As expected, the overexpression of miR-27a, miR-29a, and miR-21 reduced the circSLC8A1's expression (Figure 3(c)). Finally, we showed that miR-27a and miR-29a were downregulated in PCa (Figure 3(d)); however, miR-21 was upregulated in PCa, and miR-133b and miR-373 were not differently expressed between PCa and normal tissues. These results indicated that miR-21 might directly interact with circSLC8A1 in PCa and was selected for further validation.

### 3.4. CircSLC8A1 Suppressed PCa Progression through Targeting miR-21

**Figure 4:**
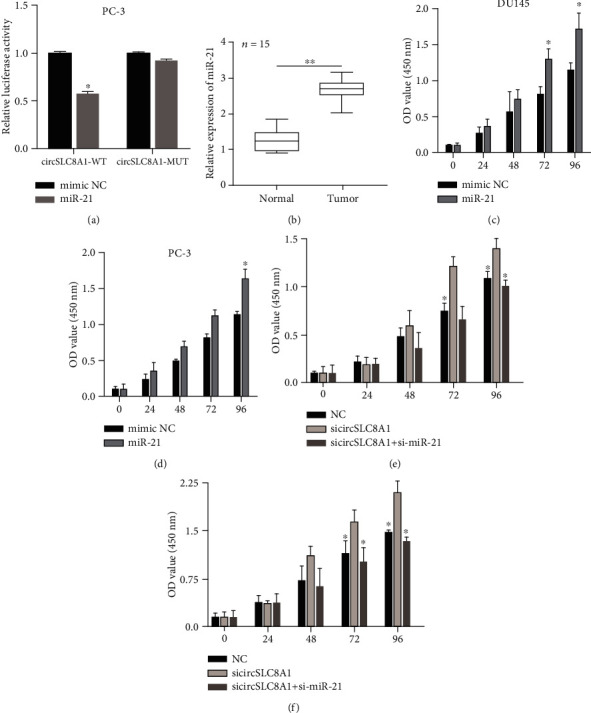
circSLC8A1 suppressed prostate cancer progression through targeting miR-21. (a) miR-21 mimics significantly reduced the luciferase of wild type circSLC8A1. (b) miR-21 was upregulated in the prostate cancer tissue. (c, d) miR-21 mimics significantly promoted the proliferation of DU145 and PC-3. (e, f) Knockdown of circSLC8A1 enhanced the proliferation ability, but this effect can be partially attenuated by the knockdown of miR-21.

To confirm to the interaction between miR-21 and circSLC8A1, we conducted luciferase reporter gene analysis. miR-21 mimics could significantly reduce the luciferase of wild type circSLC8A1 compared with mimic NC (Figure 4(a)). We next evaluated the miR-21 potential functional role in prostate cancer. The qRT-PCR results showed that miR-21 was upregulated in the PCa tissue as compared with the normal prostate tissue (Figure 4(b)). Notably, the enhanced expression of miR-21 promoted the proliferation of DU145 and PC-3 (Figures 4(c) and 4(d)), suggesting that miR-21 might be a tumor-promoting miRNA. In order to evaluate whether circSLC8A1 inhibits the progression of PCa through miR-21, we cotransfected sicircSLC8A1 and miR-21 inhibitor mimics into PCa cells. CCK-8 experiment showed that knockdown of circSLC8A1 could enhance the proliferation ability, while this enhance can be partially attenuated by the knockdown of miR-21 (Figures 4(e) and 4(f)). These results indicated that circSLC8A1 could inhibit the progression of prostate cancer through sponging miR-21.

### 3.5. The Downstream Pathways of the circSLC8A1/miR-21 Axis

**Figure 5:**
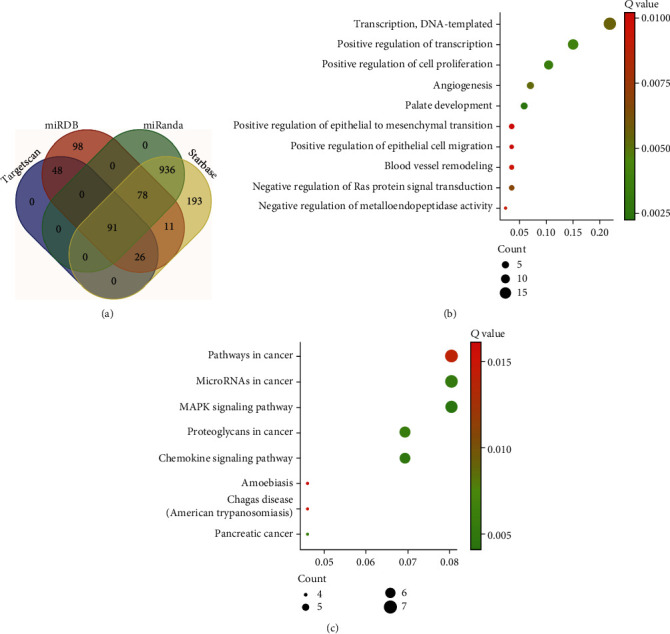
Bioinformatic analysis of the circSLC8A1/miR-21 axis. (a) Identification of targets of miR-21 by analyzing TargetScan, miRDB, miRanda, and Starbase. (c, d) GO analysis and KEGG pathway analysis of the 91 genes.

To further investigate the potential mechanism of the circSLC8A1/miR-21 axis, we searched for the target genes of miR-21 using four miRNA-mRNA interaction databases, including TargetScan, miRDB, miRanda, and Starbase. Totally, we identified 91 genes which had the potential to interact with miR-21 in PCa (Figure 5(a)). The Gene Ontology (GO) enrichment analysis showed that circSLC8A1/miR-21 was related to the regulation of cell proliferation, angiogenesis, cell migration, and epithelial to mesenchymal transition Figure 5(b)). KEGG pathway analysis showed that circSLC8A1/miR-21 was related to the MAPK signaling pathway and chemokine signaling pathway (Figure 5(c)). These results indicated that circSLC8A1 might act as a tumor suppressor by regulating cancer-promoting pathways via miR-21, thereby inhibiting cell growth and progression in PCa.

## 4. Discussion

Prostate cancer (PCa) is the second most commonly diagnosed cancer in men worldwide. Here, for the first time, we identified circSLC8A1 as a key circRNA, which is frequently decreased in PCa. The PCa patients with high circSLC8A1 expression had significantly longer overall survival than those with low expression. Our data demonstrated that circSLC8A1 could restrain PCa progression by sponging miR-21.

CircSLC8A1 is a new circRNA that has been found to be related to the progression of a variety of human benign diseases, such as osteoporosis [25], Parkinson's disease (PD) [26], and dilated cardiomyopathy (DCM) [27]. For instance, circ-SLC8A1 modulates osteoporosis via occluding the inhibited impacts of miR-516b-5p on the expression of AKAP2 [25]. Large number of independent reports have suggested that circSLC8A1 plays essential role in cardiomyocytes. Cytoplasmically localized circSLC8A1 can interact with miRNA133a-3p. Blockade of circSLC8A1 reduces the release of active oxygen by ischemia/reperfusion during the deterious effects of reactive oxygen in vivo and in turn was decreased by CDIP1, an apoptosis promoter gene to inhibit cardiomyocyte apoptosis [28]. Very interestingly, a recent study showed that circSLC8A1 is implicated in tumorigenesis. For example, Lu et al. reported that circSLC8A1 curbed PCa progression via modulating PTEN [29]. circSLC8A1 is reduced in the tissues and cell lines of PCa, and its expression is associated with the pathological and histological stages of PCa. The overexpression of circSLC8A1 resulted in the reduction of cell migration, invasion, and proliferation [29]. In mechanism, we found that circSLC8A1 directly interacted with miR-21 and consequently acted as a sponge of miRNA to modulate miR-21, and we found that miR-21 could be phagocytosed by circSLC8A1. Small RNA-21 (miR-21), as a solid body neoplasm, commonly upregulated miRNA. miR-21 has been found to participate in cell proliferation, migration, invasion, apoptosis, and drug resistance [30]. Several target genes of miR-21 have been determined, including PDCD4, SPRY2, PTEN, RECK, TPM1, and BCL2 [31]. The present study showed that miR-21 was upregulated in PCa tissue and exhibited a negative correlation with circSLC8A1. The high miR-21 expression was widely reported by previous studies [32, 33] and could significantly induce DU145 and PC-3 cell proliferation. Moreover, our data suggested that circSLC8A1 mediated that the tumor-suppressing effect could be weakened by overexpressing miR-21.

In mechanism, we predicted the downstream pathways regulated by the circSLC8A1/miR-21 axis. Specifically, circSLC8A1/miR-21 was associated with the GO terms such as regulation of cell proliferation, cell migration, and KEGG pathways such as MAPK signaling pathway and chemokine signaling pathway, indicating that circSLC8A1 might act as a tumor suppressor by regulating cancer-promoting pathways via miR-21, thereby inhibiting cell growth and progression in PCa.

## 5. Conclusions

In conclusion, our results showed that circSLC8A1 was downregulated in PCa and could function as a sponge of miR-21. In addition, we also demonstrated that circSLC8A1 inhibited PCa progression through targeting miR-21 and might serve as a novel biomarker for the treatment of PCa.

## Data Availability

All the raw data can be provided if any qualified researchers required.
